# Ecological Design of New Efficient Energy-Performance Construction Materials with Rigid Polyurethane Foam Waste

**DOI:** 10.3390/polym12051048

**Published:** 2020-05-03

**Authors:** Raúl Briones-Llorente, Ricardo Barbosa, Manuela Almeida, Eduardo Atanasio Montero García, Ángel Rodríguez Saiz

**Affiliations:** 1 Department of Electromechanical Engineering, University of Burgos, Avenida de Cantabria s/n, 09006 Burgos, Spain; rbriones@ubu.es (R.B.-L.); emontero@ubu.es (E.A.M.G.); 2 Department of Civil Engineering, University of Minho, Campus Azurém, 4800-058 Guimarães, Portugal; ricardobarbosa@civil.uminho.pt (R.B.); malmeida@civil.uminho.pt (M.A.); 3 Department of Architectonic Constructions, University of Burgos, Calle Villadiego s/n, 09001 Burgos, Spain

**Keywords:** computer simulation, ecological mortar, energy efficiency, polyurethane waste, prefabricated, slag

## Abstract

An ecological mortar is designed from industrial sub-products, with the objective of utilizing both the slag residues, generated during steel manufacturing processes, and the waste from Polyurethane Foam (PF) panels, generated during refrigerator chamber manufacturing processes. The ecological mortar design involves the dosing of Electric Arc Furnace (EAF) slag, together with finely ground Polyurethane Foam, cement, and additives. An energy efficient prefabricated block is designed with the mortar, for use in construction, and its energy performance is assessed as a material inserted within the envelope of a service sector (hospital) building, either as an exterior skin, or as an enclosing component within the façade interior. The main contribution of this research is the characterization of the thermo-physical and mechanical properties of a new prefabricated panel made with recycled materials. The full characterization of the properties of these new materials is presented and discussed. The new prefabricated panel demonstrates adequate thermo-mechanical characteristics as a substitute for traditional materials, while improving the sustainability of the building. As a secondary objective, the energy behaviour of the new panels when integrated in a real building is presented by means of a case study simulation. The use of computational thermal simulation confirmed that the properties of the prefabricated block influenced the annual thermal demand of the building for heating and cooling. Improvements to the thermal inertia of the building envelope were also confirmed with the inclusion of PF waste, giving the mortar an energy performance that was similar to conventional materials, in such a way that its use in façade construction may be validated, in addition to its environmental benefits, due to it having been manufactured with critical recycled industrial waste such as EAF slag and PF, thereby contributing to both the circular economy and sustainable development.

## 1. Introduction

At present, we are witnessing a new social revolution that seeks to raise awareness of the climatic changes that are happening on our planet and the danger that this implies regarding diversity [1,2,3]. New social models are attempting to seek responses to traditional industrial development, avoiding environmental impacts that contaminate and pollute, and minimizing the effects of global warming and the over-exploitation of natural resources [4,5,6]. In striving to avoid the collapse of our civilization, effort must be invested in reducing our dependency on traditional raw materials, and seeking valid alternatives through the recycling and recovery of waste for industrial and scientific progress [7,8,9].

One technological innovation in construction has been the design of lightweight mortars for use in the manufacture of new construction materials, a circumstance that can substantially reduce the loads of structures and which contributes to the insulation of buildings [10,11,12,13]. In this sense, the use of PU waste for the manufacture of these lightweight mortars is one of the research lines developed over recent years within Building Material Research Groups, attempting to find a positive compatibility between the traditional components of mortars, and the Polyurethane Foam (PF) that is generated in industrial processes, such as the manufacture of automobiles and refrigeration installations [14,15,16]. In this way, a lightweight construction material can be produced that meets the minimum requirements for mechanical strength and that, at the same time, contributes to improving the energy conditions of buildings, because of its insulative properties, with low thermal and acoustic conductivity [17,18].

The investigation developed in this paper seeks to incorporate different polymer types used in industrial processing in the manufacture of construction products, integrating them as an extra component in the dosing of concretes, mortars, and plaster pastes, among others. Numerous authors have studied interactions between polymeric products and inorganic binders, with the objective of establishing their effects on the properties of the final products. Models have been proposed, in an attempt to explain the formation of paste microstructures, as well as their interactions with other components, such as aggregates and additives [19,20,21].

Likewise, as an alternative to the use of traditional aggregate for construction, research work has also been developed to recover industrial waste with similar properties, such as steel slag generated during the steel manufacturing process [22,23,24]. Traditionally, slags have been dumped in landfill sites with no defined use, with inevitable impacts on the landscape and on the ground upon which they accumulate. These wastes present similar properties to conventional aggregates and, in some cases, because of their nature and composition, show both hydraulic and pozzolanic behavior [25,26]. Slag from a Basic Oxygen Furnace (BOF) convertor has been used for the construction of roads and bituminous pavements [27,28,29,30]. Mortars and concretes with good mechanical properties and durability have been designed with Electric Arc Furnace (EAF) black slag [31,32,33,34,35]. Finally, white slag from the Ladle Furnace (LF) has been successfully used for the manufacture of Portland cement [36], as a fine aggregate in masonry mortars [37,38], and for the stabilization of expansive clayey soils [39,40].

Moreover, it is increasingly common in building to use prefabricated materials with insulative properties that, in turn, integrate recovered waste materials [41,42,43,44,45,46,47,48,49], thereby complying with European Directives on the energy performance of buildings [50] and on waste recycling, reutilization, and recovery [51]. Accordingly, the research developed within this study proposes the design of a utility construction model (a prefabricated block), using an ecological cement mortar that incorporates doses of both Polyurethane Foam (PF) waste from the fridge chamber manufacturing industry and Electric Arc Furnace (EAF) slag, reused as aggregate, from the steel manufacturing industry. Subsequently, its applicability to the industrialized construction of exterior walls is studied by testing its thermal performance.

With this work, the main aim is to achieve an ecological material that is respectful of the environment, manufactured with recycled and recovered materials and efficient from an energy point of view when incorporating recovered PF waste material, in coherence with the provisions of the European Directives on energy efficiency and waste recycling.

A secondary objective is testing how these new ecological materials contribute to comfort levels within buildings. Then, its energy performance as a construction material placed within the building envelope is simulated. In this case study, a service sector hospital building was selected, as these buildings consume energy on a large scale in the European Union, together with large hotels and commercial centres. Their specific energy consumption varies between 250 and 600 kWh/m^2^, depending on the type of hospital, its size, its location and, of course, the state of its envelope and air-conditioning systems [52]. The comparison of some studies from the 1980s and 1990s [53,54,55] with other more recent ones [56,57], including projects financed by the EU (RES-Hospitals, LCB Healthcare, Green hospitals, etc.), indicated that energy consumption in buildings has not significantly fallen, at least not in proportion to theoretical advances concerning envelopes and air-conditioning systems, and the implementation of the European Directive on the Energy Performance of Buildings (EDEPB) [58] after 2003.

## 2. Materials and Methods

The experimentation process includes two large sections. First, the study of the properties of the mortar used to manufacture the precast panel. Second, the simulation process using specific software to check its energy behavior when integrated on the facade of a building.

### 2.1. Utility Model Design

The objective of this research work is the design of a new ecological construction material, with a good technical performance, for use as an energy-efficient material in building enclosures.

**Figure 1:**
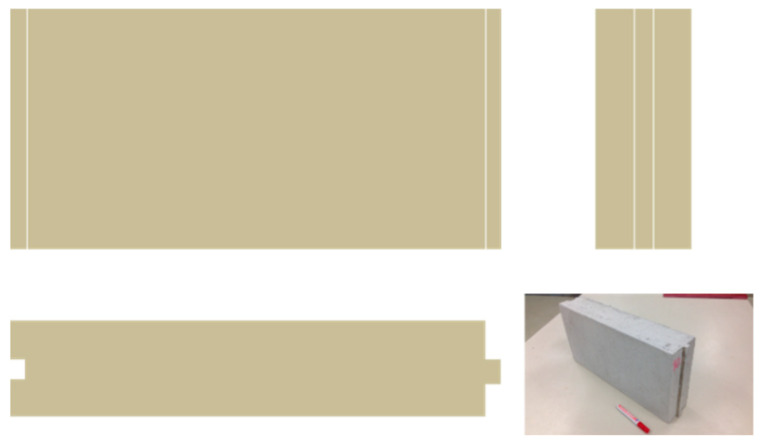
Ecological cement mortar block.

The utility model that is designed is a tongue-and-groove block, with dimensions of 500.0 × 250.0 × 100.0 mm, manufactured with the ecological mortar composed of cement, recovered steel slag, recycled PF waste, a suitable additive, and mix water. The design of the utility model and its geometry may be seen in Figure 1.

### 2.2. Materials

An ecological cement mortar was designed for the manufacture of the Utility Model, containing recovered industrial waste that adhered to the specifications of European standard EN 998-2:2018 Specification for mortar for masonry—Part 2: Masonry mortar [59] as a reference.

The basic materials used in the ecological mortar mix design for molding the prefabricated monolithic block were as follows:

Portland Cement CEM I 42.5 R was manufactured by the firm Cementos Portland Valderribas at its factory in Mataporquera (Cantabria, Spain), in accordance with the specifications of European standard EN 197-1:2011 [60]. Due to its characteristics, this cement is ideal for the preparation of prefabricated components, because it is composed of 95% Portland cement clinker and 5% lime. Its Blaine specific surface, 3400 cm^2^/g, enables a rapid hydration and an ideal pouring time that facilitates the molding of each piece. Its principal characteristics are shown in Table 1.

**Figure 2:**
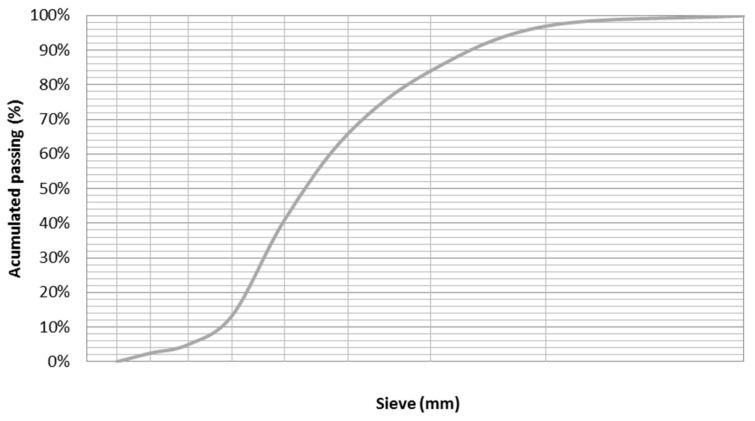
Granulometry Electric Arc Furnace Slag (EAF).

Electric Arc Furnace (EAF) black slag is an industrial sub-product from steel manufacturing. This waste had previously been weathered under laboratory conditions to stabilize the expansive components—Calcium Oxide (CaO) and Magnesium Oxide (MgO)—by means of hydration, favouring its transformation into Portlandite (Ca(OH)_2_) and brucite (Mg(OH)_2_). Sieve sizes smaller than 8.0 mm. were selected and 97% of the slag was smaller than 4.0 mm, so it was designated as an arid Ø 0–4 mm, in accordance with European standard EN 13139:2002/AC: 2004 [61]. Its granulometric distribution is shown in Figure 2.

The slag, once stabilized, was analyzed with X-ray Fluorescence Spectroscopy, with a Thermo Electron Corporation ARL ADVAT XP Sequential XRF with Claisse Fluxy. The most important components identified from the analysis are shown in Table 2.

**Figure 3:**
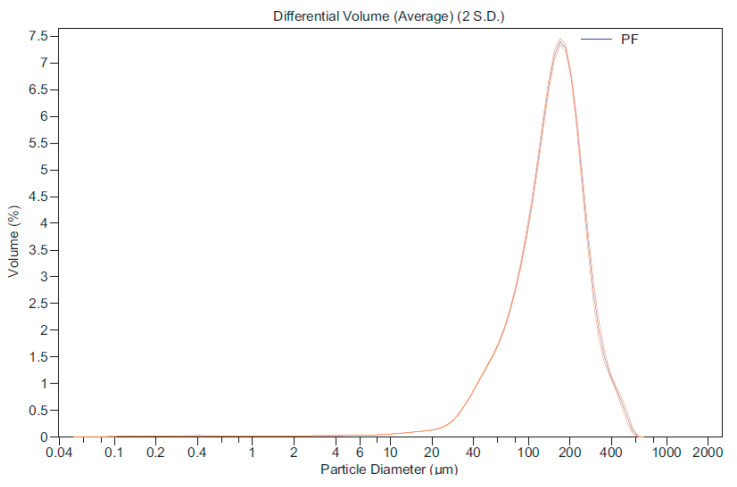
Granulated rigid polyurethane foam waste (PF).

Polyurethane Foam (PF) is a waste sub-product from the cutting of sandwich-type panels fitted as thermal insulation inside refrigeration chambers for food transport, storage, conservation, and distribution. The foam was reduced in size by cutting, using an SM 100 RETSCH cutting mill. Subsequently, a granulometric analysis was performed with a Beckam Coulter LS 13 320 Analyzer, yielding a particle distribution by size that is shown in Figure 3.

The chemical composition of the PF, the result of CHNS elemental analysis with a LECO CHNS-932 analyzer and X-ray diffraction, is shown in Table 3.

An air entrainer–plasticizer additive was used to reduce the surface tension between the dosed water and the mortar components, favouring its hydration. The additive, dosed at 0.8% by weight of cement, was supplied in powder form by the firm BASF.

The water used for mixing the solid components of the mortar was taken directly from the mains water supply of the Sociedad Municipal Aguas de Burgos (Burgos Municipal Water Corporation, Burgos, Spain).

### 2.3. Technical Criteria for Dosing the Mortar

The mortar was dosed in accordance with a component by volume ratio (RV) of [1:(1 + 3):1] for the components (cement/EAF + PU/water). As the ecological mortar design will be used for molding prefabricated pieces, the incorporation of the air entrainer–plasticizer additive reduced the mix water, but maintained good workability, with a slump on the flow table of 150 ± 10 mm. The components of the mixture are shown in Table 4.

### 2.4. Properties and Features of Mortars

The ecological design mortar was characterized in accordance with the specifications of European standard EN 998-2:2016 [54]. Table 5 shows a summary of the different test results.

#### 2.4.1. Density and Air Content of Fresh Mortar

Fresh and hardened density and occluded air were measured at a temperature of 20 ± 1 °C and a relative humidity of 50 ± 1%, according to European Standards EN 1015-6 and EN 1015-7 [62,63].

#### 2.4.2. Dry Bulk Density of Hardened Mortar

The dry bulk density of the hardened mortar was determined in accordance with the specifications of European standard EN-1015-10 [64]. In accordance with its composition, the density of the hardened mortar was 1321.40 kg/m^3^, in other words, it presented a low density.

#### 2.4.3. Mechanical Properties: Flexion, Compression and Adherence

Specimens prepared in molds, measuring 40 mm × 40 mm × 160 mm, were cured at 20 °C and 98% relative humidity, in order to determine the flexural and compressive strength of the mortar, as per standard EN 1015-11 [65]. The samples were tested both at 7 and at 28 days of age and three flexural tests were performed, with a separation of the supporting rollers of 100 mm. The resulting fragments were subjected to six compressive strength tests performed on a surface area of (40 × 40) mm.

The compressive strengths of the different mortars at 28 days (3.85 N/mm^2^) are shown below in Table 4, hence its classification as M-2.5.

**Figure 4:**
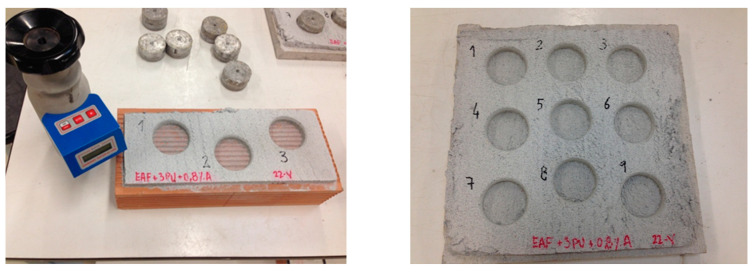
Determination of adhesive strength of hardened rendering and plastering mortars on substrates.

The resistance to adherence was determined in accordance with European standard EN 1015-12 [66], taking as a reference a porous ceramic surface and a ceramic tile manufactured from the same material, as shown in Figure 4. The results on the ceramic surface (0.11 N/mm^2^) and the mortar tile (0.27 N/mm^2^) indicated that the design mortar easily adhered to both surfaces.

#### 2.4.4. Determination of Water Absorption Coefficient in Hardened Mortar

**Figure 5:**
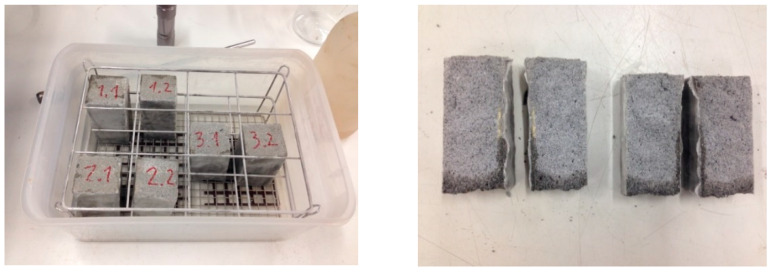
Determination of water absorption coefficient in hardened mortar.

The determination of the water absorption coefficient, due to the capillary action of hardened mortar, was performed in accordance with the test specified in European standard EN 1015-18 [67], applied to six standardized specimens of 40 mm × 40 mm × 160 m. Likewise, the height of the water, drawn upwards by capillarity action, was determined as shown in Figure 5.

In accordance with European standard EN 1015-18 [67], the classification of the Capillarity Absorption Coefficient (c = 0.2083 Kg/(m^2^·min^0.5^) value of the mortar corresponded to W2 (c ≤ 0.2 Kg/(m^2^·min^0.5^), the water ascending to an average height of 10.0 mm.

#### 2.4.5. Determination of Water Vapour Permeability

**Figure 6:**
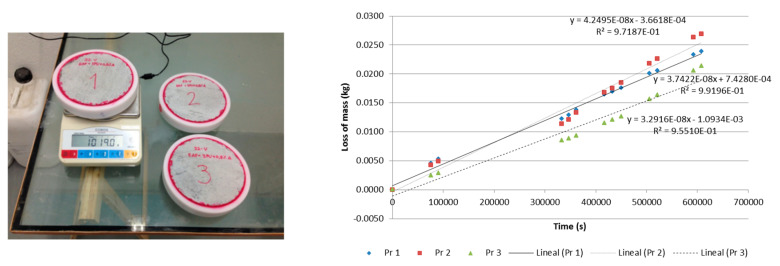
Determination of water vapour permeability.

The water vapour permeability level of the mortar was determined by European standard EN 1015-19 [68]. To do so, three cylindrical specimens were molded and each one was sealed within a mold with a saturated saline solution inside. The recipients holding the specimens were maintained in a water temperature-controlled environment and under a constant water pressure, differing from the interior pressure. By observing the weight variation in the two items, recipient and specimen, under long-term test conditions, the moisture vapour transmission rate through the mortar, shown in Figure 6, was determined.

The mean value of the water vapour permeability of the three test specimens tested was 4.30564 × 10^−11^ kg/m·s·Pa, and the Water Vapour Diffusion Resistance Factor (µ) is 4.

#### 2.4.6. Determination of Water Absorption at Atmospheric Pressure

As no specific regulation exists on mortar water absorption at atmospheric pressure, the procedure for natural rocks established in standard EN 13755 [69] was used and can be partially justified by the stony nature of the mortar texture.

To perform the test, three cubic specimens of 50 mm × 50 mm × 50 mm were used, taking the average absorption of the three specimens as a reference, as shown in Table 6. The absorption of water at atmospheric pressure is expressed as the percentage weight of absorbed water.

#### 2.4.7. Determination of Specific Heat of Mortar

The specific heat (Cp) of the mortar with foams was determined in the Applied Physics Laboratory of the University of Burgos, using a High-Temperature Differential Scanning Calorimeter, applying the Mixture Method (obtaining the determination of specific heat by means of a comparative method and standard samples). The experimental test results yielded a specific heat of 1291.76 J/kgK. for the ecological mortar design.

#### 2.4.8. Determination of Thermal Conduction Coefficient

Mortar tiles with dimensions of 30 mm × 30 mm × 2 mm were manufactured and tested with the procedure established in European standard EN 12664 [70]. The test results are shown in Table 7.

### 2.5. Energy Simulation of the Building

The energy behaviour of the mortar block as a constructive element integrated within a real building in the two proposed types of façade simulation was studied and compared with the existing façade. The tests are useful for establishing whether this new material behaves at least equally to the conventional materials which it may replace.

The annual energy demand of the heating and cooling system on two storeys that are representative of the proposed building was studied, supposing that each of the three types of façades were installed. The three case studies proposed for each of the two storeys of the building were compared, thereby contributing information on the thermal behavior of this new construction material, which complements the hygro-thermal characterization, the condensation study, and the thermal inertia tests previously carried out.

As indicated earlier, the energy simulation was performed with a Transient System Simulation (TRNSYS v.17, Thermal Energy System Specialists, LLC, Madison, WI, USA) software package [71], an extremely flexible graphic-environment based software package that is used to simulate energy flows within transitory systems, such as buildings. It consists of two parts: the first processes the input data, computes the system by iteration, determines convergence values, and lists the system variables. The second part is an extensive component library of use for modeling the functioning of any one part of the system.

The chosen case of study is the University Hospital of Burgos (HUBU), situated in Burgos, in the North of Spain. It was inaugurated in 2012, making it a good example of a modern hospital. Burgos has a similar continental climate to other central European cities.

Beginning with the composition of the actual façade of the building, the behavior of the material is studied in the form of a block for masonry constructions, under two scenarios: (i) substituting the exterior layer of the existing façade, as a visible component; and, (ii) substituting the innermost layer of the façade, with no interior finishes.

The criterion of at least not worsening and whenever possible improving the thermal behavior of these two new façade options is tested with respect to the existing façade. In the first phase, thermal transmittance, the risk of surface and interstitial condensation, and thermal inertia is studied on all three façades.

In the second phase, the influence of using each façade option on the annual thermal demand for heating and for cooling of the building is studied. The study involves a computational thermal simulation of two representative storeys with two different uses within the building.

The main objective of this research is the characterization of the thermo-physical and mechanical properties of a new prefabricated panel made with recycled materials. The first part of the article is devoted to the precise characterization of the properties of these new materials in accordance with European standards. A detailed description of the experimental techniques and the obtained results are shown. Then, as a secondary objective, the energy behaviour of the new panels when integrated in a real building, is presented by means of a case study simulation in the second part of the article.

#### 2.5.1. Building Geometry

**Figure 7:**
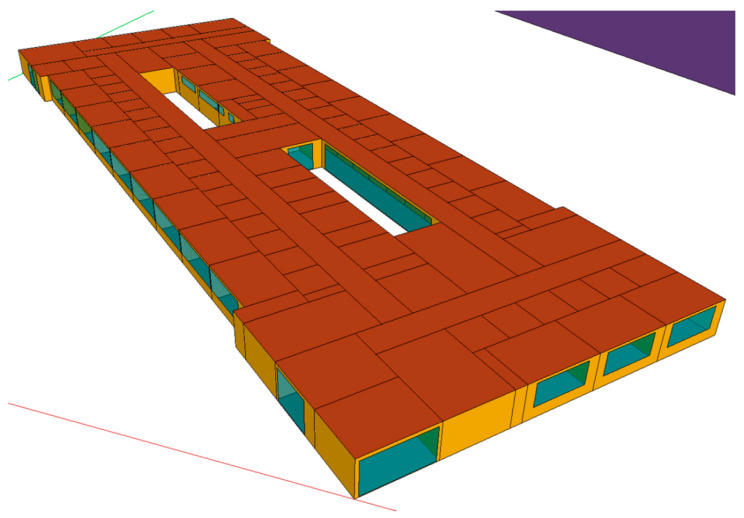
Transient System Simulation (TRNSYS) energy simulation of the hospital inpatient ward floor.

**Figure 8:**
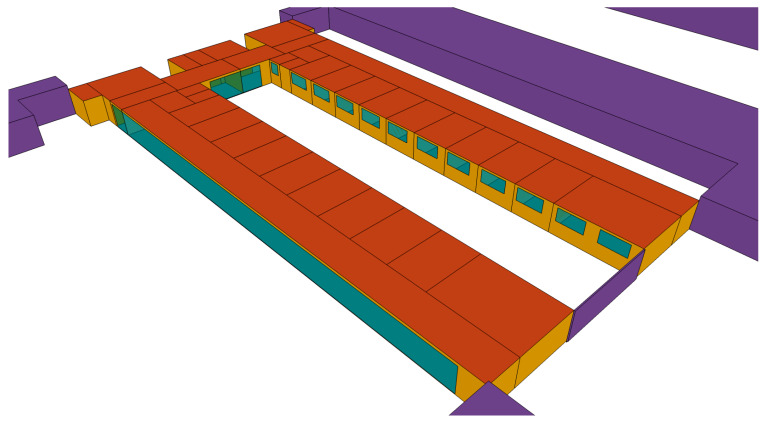
TRNSYS energy simulation of the hospital outpatient consultation floor.

Two storeys of two representative areas with different uses within the hospital were simulated. The simulations completed with TRNSYS [71] are shown in Figure 7 and Figure 8.

The hospital inpatient ward floor measures 72.85 m in length and 26.36 m in maximum width, measured from the exterior wall. The free-standing interior height is 2.70 m. It is not situated on the ground, but is raised on piles over three storeys.

The hospital outpatient consultation floor measures 50.75 m in length and has a maximum width of 27.38 m, measured from the exterior. The interior free-standing height was 2.70 m. It is the third storey of the building, below the hospital inpatient ward floor, that is also studied in this paper.

**Figure 9:**
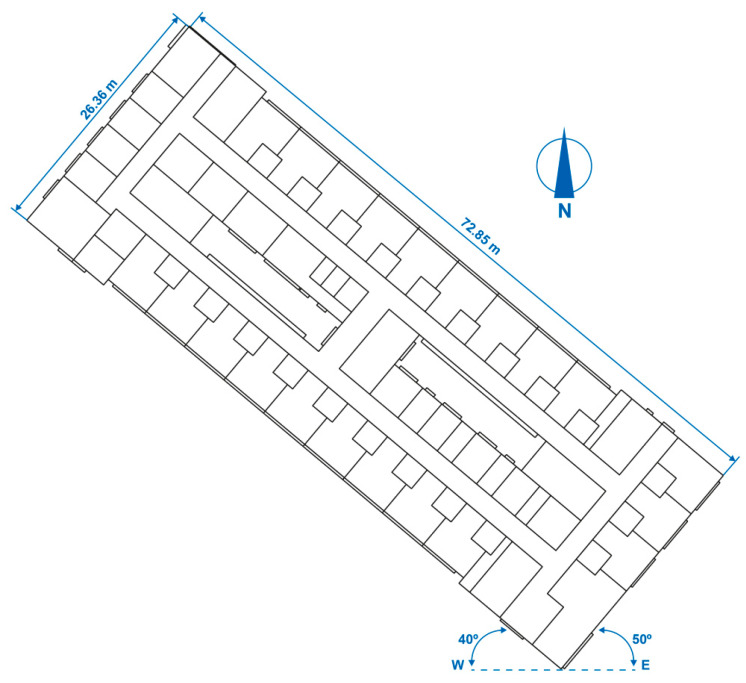
Geometry of the hospital inpatient ward floor.

**Figure 10:**
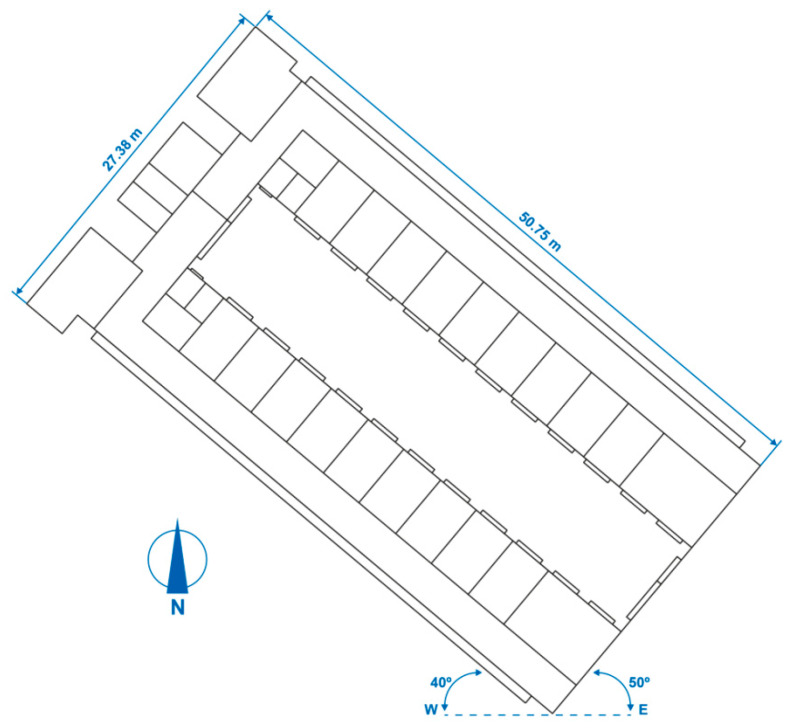
Geometry of the hospital outpatient consultation floor.

Both floors are offset from the east–west direction. The longest façades are rotated by 40° and the shortest façades are rotated by 50°, both along an east–west axis, as shown in Figure 9 and Figure 10.

#### 2.5.2. Constructive Characteristics of the Building

The exterior enclosures and the interior partitions were fitted in layers and are in agreement with TRNSYS [71], taking into account the physical characteristics of the materials of each layer.

The convective heat-transfer coefficients of the building enclosures are detailed in Table 8, and are in agreement with the Basic Document DA DB-HE/1, of the Spanish Building Code [72].

The solar absorptance values of the building enclosures are detailed in Table 9, and are in agreement with DA DB-HE/1 [72].

All the thermal bridges on both floors of the building were identified and processed for display with DA DB-HE/2 [73]. The values of their linear thermal transmittance are detailed in Table 10, and are in agreement with the Spanish Building Code [74].

Double-glazed windows were used with a sealed air chamber (4/15/4 mm) and an aluminium frame with a thermal break. Their characteristics are shown in Table 11.

#### 2.5.3. Operational Conditions of the Building

The hospital inpatient ward floor is busy on all days of the week. Patients are admitted who are receiving medical treatment and, likewise, receive visits from family and friends. There are no high internal heat gains due to the occupancy levels, lighting, and equipment.

The outpatient consultation floor is occupied from Monday to Friday, but only in the mornings. It contains the consulting rooms where the doctors receive the patients for examination and discussion of their possible illnesses and treatments. There are moderate internal heat gains due to the level of occupancy, but low gains with regard to lighting and equipment.

The two user profiles were generated with TRNSYS [71], using as a starting point the profiles that appear in the Spanish Building Codes [74] “Non-residential use over 24 h of low intensity” and “Non-residential use over 8 h of average intensity”, respectively, and taking into account the information supplied by the managers of the hospital. The internal heat gains due to lighting were calculated considering the 80 lm/W compact fluorescent lighting and the average horizontal illuminance, which was 500 lux.

The numerical values that defined both user profiles are shown in Table 12, Table 13, Table 14, Table 15, Table 16 and Table 17.

The definition of the air-conditioning system is not among the objectives of this investigative work.

#### 2.5.4. Climatic Conditions

The hospital under study is situated in the city of Burgos, in the north of Spain. It is on a latitude of 42°17′10″ north and its longitude is 3°27′22″ west. Its height above sea level is 861 m. This city has one of the severest winter climates in Spain, according to the Spanish Building Code [74]. The average monthly temperature is shown in Table 18.

### 2.6. Hygro-Thermal Behaviour of the Mortar Block

**Figure 11:**
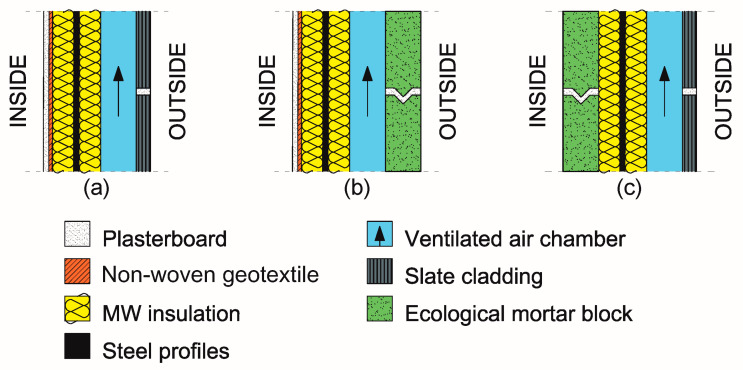
Constructive sections of the façades. (**a**) Current façade. (**b**) Façade with the ecological mortar block outward. (**c**) Façade with the ecological mortar block inward.

Exterior enclosures and interior partitions of the building were generated layer-by-layer, from the interior toward the exterior, with the Transient System Simulation (TRNSYS v.17, Thermal Energy System Specialists, LLC, Madison, WI, USA) software package [71]. The geometric and thermophysical characteristics of the three facades are shown in Table 19, Table 20 and Table 21. Some constructive sections are depicted in Figure 11.

The general characteristics of the three façades are shown below, in Table 22.

### 2.7. Surface and Interstitial Condensation

Subsequently, the risk of superficial and interstitial condensation was studied on each of the three façades under study. The program eCondensa2 [75], implementing the calculation method that can be found in DA DB-HE/2 [73], forms part of the Spanish Building Code [74], which is, in turn, the Spanish transposition of the European Directive [58]. 

These values of superficial and interstitial condensation were calculated for the city of Burgos in January, the month in which the exterior conditions were least favourable (2.6 °C) and the relative humidity was high (86.0%). The interior conditions were: temperature (20.0 °C) and relative humidity (55.0%).

### 2.8. Thermal Inertia

The thermal inertia variations of the existing façade were studied, having changed their composition by fitting exterior mortar blocks and interior mortar blocks, as is detailed in Table 19, Table 20 and Table 21, respectively.

When the mortar blocks are fitted to the exterior, they replace the slate cladding, and are thicker and have a higher specific heat. They are placed over the thermal insulation layer, facing outwards.

When the mortar blocks are placed within the interior, they replace the laminated gypsum panels and non-woven geotextile. Their greater thickness and density mean that they have a higher specific heat than the laminated gypsum panel, but a lower specific weight than the non-woven geotextile. They are placed under the thermal insulation, towards the interior.

Five magnitudes were calculated in relation to the thermal inertia for the three façades that were proposed. Thermal inertia (1) is the capability of a material to store energy that depends on its mass, its density, and its specific heat. The materials with higher thermal inertia will take longer to reach thermal equilibrium with the surrounding media when a thermal gap exists between both:

*I* = (*λ × δ × C_p_*)^1/2^, J/(m^2^ × K × s^1/2^) (1)

The second magnitude that will be studied is thermal mass (2), which is the quantity of heat that a body is capable of absorbing and storing:

*m_t_ = δ × C_p_ × t*, J/(m^2^ × K) (2)

The third magnitude is the thermal constant (3), which shows the reaction time of a body following changes to the exterior temperature:

*CTT = [R_se_ + (0.5 × R*_1_*)] × m_t1_ + [R_se_ + R*_1_*+ (0.5 × R*_2_*)] × m_t_*_2_*+ … + [R_se_ + R*_1_*+ R*_2_*+ … + (0.5 × R_n_)] × m_tn_, s* (3)

The fourth magnitude is the useful thermal mass (4), which is the thermal mass of the construction materials placed between the layer of thermal insulation and the interior, and which is capable of accumulating energy:

*m_tu_ = CTT/R_T_, J*/(m^2^ × K) (4)

The fifth magnitude is the useful thermal mass percentage (5), which is the relationship between the useful thermal mass and the total thermal mass of the façade:

% *m_tu_ = m_tu_/m_t_,*% (5)

## 3. Results and Discussion

### 3.1. Utility Model Designed

The apparent density of the in-fresh mortar was determined with the test specifications from European standard EN 1015-6, obtaining a value of 1321.4 kg/m^3^. This density produces a material with a low slump (150 mm), but can be easily placed due to the incorporation of the air entrainer–plasticizer, water reducer additive, which helps mold the pieces. The dry plastic consistency of the mortar was adjusted, to meet the specifications of European standard EN 1015-6 [62], in other words, with a consistency of 160 ± 10 mm.

An air occlusion value of 37% was achieved in the mortar with the air entrainer–plasticizer additive, giving it an extensive capillary network through which water vapour can transpire, thereby preventing any possible accumulation of humidity in the internal zones of the building enclosures. A behavior that the water vapour permeability test results confirmed where the resistance factor, µ, to water vapour diffusion was µ = 5, making it a mortar that permitted the water vapour to pass through it quite easily.

Nevertheless, once the capillarity water absorption test was over, although the capillary network was large, it was seen that the pores were not sufficiently wide for the water to rise though the interstitial network of the mortar (Jurin’s Law). In accordance with European standard EN 1015-18 [67], the Water Absorption Coefficient by capillarity value (c = 0.2083 Kg/(m^2^·min^0.5^) graded the mortar as W2, quite an impermeable mortar, with good resistance to water filtration—properties which are proven by the average height of the ascension of the water, which was only 10 mm.

### 3.2. Surface and Interstitial Condensations

**Figure 12:**
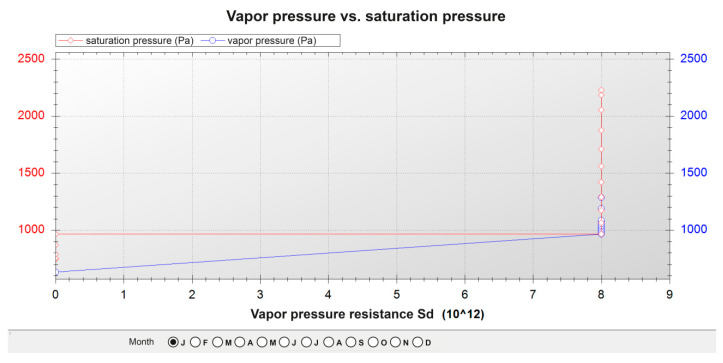
Pressure graph of the current façade.

**Figure 13:**
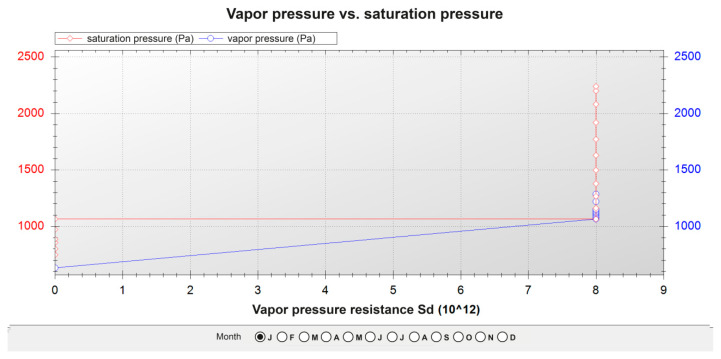
Pressure graph of the façade with the ecological mortar block outward.

**Figure 14:**
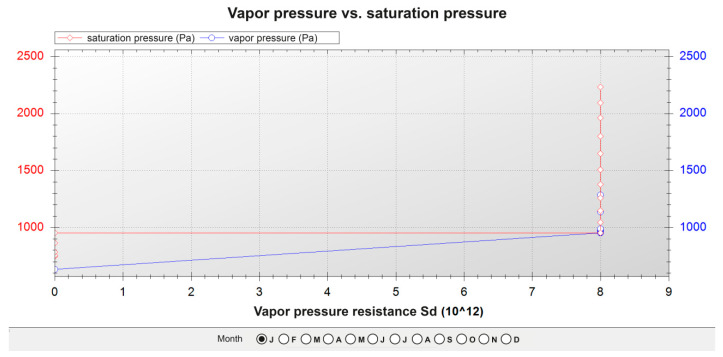
Pressure graph of the façade with the ecological mortar block inward.

Table 23, Table 24 and Table 25 show the results of the calculations for the three façades. The layers of the different materials that form the façades were ordered, from the exterior to the interior, to be able to compute those calculations. The graphs of water pressure against saturation pressure for the three façades throughout the month of January are shown in Figure 12, Figure 13 and Figure 14.

In the three cases:

- There is no risk of surface condensation;
- There is no condensation on the thermal insulation layers;
- There is an accumulation of condensation on the layers of steel profiles, but in the annual balance, the accumulated amount of condensation was inferior to the evaporation levels.

### 3.3. Thermal Inertia

Table 26 shows the results of the calculations of all five thermal inertia-related magnitudes for the three façades, previously explained in Section 2.8.

With regard to the total thermal inertia of each façade, the existing one and the one with interior mortar blocks presented very similar values, and the one with the exterior mortar blocks showed a slightly lower simulated value.

The thermal mass values ordered from low to high would be as follows:

- Existing façade < façade with exterior mortar blocks < façade with the interior blocks;
- The rising values are due to the thermal mass of the mortar block layers, greater than the thermal mass of the material layers that they replaced, considering the existing façade;
- The thermal constant, the useful thermal mass, and the useful thermal mass percentage increased by the same proportions as the previous magnitude;
- The high thermal constant of the façade with the interior blocks with respect to the other two façades attracts attention. It indicates that its temperature will take much longer to vary when the interior and exterior temperatures vary. It will, for example, conserve the heat accumulated during the day for longer, which will then slowly dissipate during the night towards the interior spaces, as it is in contact with them, and the insulative thermal layer will prevent most of the thermal flow from passing toward the exterior;
- In the same sense, the useful thermal mass and the percentage of useful thermal mass are also much greater on the interior mortar block layers of the façade, because these layers add greater density, specific heat, and thickness to the insulative thermal layer toward the interior;
- The composition of the interior mortar block layer of the façade is therefore interesting in those spaces where an accumulation of heat or cold is sought in the walls, so that it subsequently dissipates towards the interior space;
- However, when the opposite is desired, and no accumulation of heat or cold is desired in the walls that can dissipate towards the interior, both the façade with exterior mortar blocks and the composition of the existing façade would be more appropriate.

### 3.4. Energy Simulation of the Building

The results of the annual energy demand for heating and cooling after completing six energy simulations of the three types of façade by means of [71] are shown in Table 27, for the hospital inpatient ward floor and for the outpatient consultations floor, respectively. The total annual results, normalized to the useful floor area, are shown to facilitate their comparison.

In an initial global analysis, and taking into account the operational conditions of the building described in Section 2.5.3, it can be seen that the hospital inpatient ward floor has (approximately) a 50% higher heating demand than the hospital outpatient consultations floor. Taking into account that the constructive characteristics are similar, and that they have the same orientation, this may be due to the hospital inpatient ward floor having three times the daily hours of use of the hospital outpatient consultations floor, and also half of the internal loads that imply free heating.

In contrast, the hospital outpatient consultations floor has (approximately) a 10% higher cooling demand than the hospital inpatient ward floor. Despite the shorter period of utilization, this higher demand may be due to the higher quantity of internal loads, and because, outside the period of use, the ventilation will not be functioning. There will therefore be no free cooling during nocturnal hours, which is especially necessary in the summertime, as the hospital inpatient ward floor has this characteristic.

The energy heating demand was, approximately, eleven and seven times higher than the cooling demand of the hospital inpatient ward floor and the hospital outpatient consultations floor, respectively. This higher demand was due to the harsher conditions of the winter climate simulated for the city of Burgos, in comparison with those of summer, as explained in Section 2.5.4.

It can be seen from the analysis of the constructive compositions of the three façades that their results are very similar. These results are due to the very similar thermal transmittance values of the three enclosures, as may be seen in Table 22. The ecological mortar blocks represent only one component of the thermal envelope of the storeys that are under study, as a high surface area of the façades is also glazed, so any modification of opaqueness is low.

The energy heating demands, ordered from high to low, would be as follows:

- Hospital inpatient ward floor: existing façade > façade with the exterior recycled mortar blocks > façade with interior recycled mortar blocks. The latter façade, despite its higher thermal transmittance than the previous façade, is the façade with higher thermal inertia values, as may be seen from Table 26;
- Hospital outpatient consultations floor: façade with interior recycled mortar blocks > current façade > façade with the exterior recycled mortar blocks. Here, too, the thermal inertia values may be of greater influence than the thermal transmittance values.

The energy demands of cooling, ordered from highest to lowest, would be as follows:

- Hospital inpatient ward floor: existing façade > façade with the exterior recycled mortar blocks > façade with the interior recycled mortar blocks. The same was applicable to this storey as for the analysis of heating demand;
- Hospital outpatient consultations floor: façade with the exterior recycled mortar blocks > façade with the interior recycled mortar blocks > existing façade. The demand coincides with the rising values of thermal transmittance. As this floor is not in continuous use, the effect of thermal inertia will be lower;
- In any case, the three façades showed very similar energy behaviours over one year, which validated the recycled mortar blocks that were used to replace both slate as an exterior cladding, and the gypsum and cardboard panelling and non-woven geo-textiles as interior layers.

## 4. Conclusions

An ecological mortar cement has been designed with the addition of industrial Polyurethane Foam (PF) waste for use as a recovered material in the manufacture of ecological cement blocks that can be used to improve the thermal performance of constructive elements on building façades. This results in the reduced use of raw materials, which are replaced by recovered materials that will no longer be disposed of as waste materials, which implies a double saving in materials, energy and toxic environmental emissions. In addition, the positive effect that the incorporation of this recovered PF waste has on the energy performance of the design material has been confirmed.

The incorporation of the prefabricated mortar blocks designed with recovered materials has been studied on the façades of two storeys of a hospital block with different uses. The façade has been analyzed in its existing state and in two alternative cases, replacing their exterior and their interior layers with these blocks.

In a detailed comparative study of the three types of façades, it was concluded that: (i) they presented very similar thermal transmittance values; (ii) there was no risk of surface or interstitial condensation, nor of any effect on thermal insulation; (iii) an interior layer of blocks on the façade greatly improved its thermal inertia, which is desirable in the hospital inpatient ward floor in continuous use; (iv) the placement of the mortar blocks as an exterior layer implied similar thermal inertia values to the existing façade and less than in the earlier case; for this reason, a more limited use is suggested for the hospital outpatient consultations floor.

Six energy simulations have been conducted of the two storeys with the three types of façades, in order to understand the influence of each one on the annual energy behaviour of each storey. The influence of exterior climatic conditions may be appreciated, as well as for the two user profiles that have been proposed. However, energy behaviour in response to heating and cooling demand for the three façades was very similar for each of the two storeys under study.

Based on the results obtained with this detailed double analysis and the analysis of annual energy behaviour, the use of the recycled mortar blocks to replace the other conventional constructive elements within the building can be validated, as they provide similar performance levels, thereby maintaining the criteria of, at the very least, not worsening and possibly improving the performance of the building, in addition to the environmental benefits of reusing recovered industrial waste.

## Figures and Tables

**Table 1:** Technical characteristics of Portland Cement CEM I 42,5 R.

Element	Clinker	Limestone Filler	SO_3_	Cl	Ignition Loss	Insoluble Residue
% Mass	95.0	5.0	3.2	0.01	3.2	1.4

**Table 2:** Chemical composition of Electric Arc Furnace Slag (EAF).

Values	CaO	SiO_2_	Fe_2_O_3_	Al_2_O_3_	MgO	Cr_2_O_3_	MnO	P_2_O_5_	SO_3_	Others	Total
%	25.78	14.08	34.35	8.54	7.73	1.55	4.83	0.43	0.42	2.29	100.00

**Table 3:** Chemical composition of rigid polyurethane foam (PF).

Sample PU (mg)	Values	C	O	N	H	S	Others	Total
1.170	Mass (%)	54.0	4.9	7.5	11.7	0.0	21.9	100.0

**Table 4:** Ecological mortar components.

Values	CEM I 42,5R R	EAF	PU	Additive	Water
Ratio Volume	1	1	3	-	1.1
Weight (g)	600	1118.4	129.24	4.8	660

**Table 5:** Properties of the ecological mortar.

Standard	Test	Values	
**Fresh mortar**			
EN 1015-6:1999	Bulk density of fresh mortar	1321.40 kg/m^3^	
EN 1015-7:1999	Air content of fresh mortar	37.0%	
**Hardened mortar**			
EN-1015-10:1999	Dry bulk density of hardened mortar	1058.00 kg/m^3^	
EN 1015-11:2000/A1:2007	Flexural strength	7D-0.76 N/mm^2^	28D-1.55 N/mm^2^
	Compressive strength	7D-2.01 N/mm^2^	28D-3.85 N/mm^2^
EN 1015-12:2016	Adhesive strength on ceramic substrate	0.11 N/mm^2^	
	Adhesive strength on mortar substrate	0.27 N/mm^2^	
EN 1015-18:2003	Water absorption by capillarity	c = 0.2083 Kg/(m^2^·min^0.5^)	
EN 1015-19:1999	Water vapor permeability	µ = 5	
EN 13755:2008	Water absorption at atmospheric pressure	25.78%	

**Table 6:** Water absorption at atmospheric pressure.

Test	Sample			
	1	2	3	Mean
Dry Weight (g)	268.60	267.60	265.90	267.37
Saturated Weight (g)	336.40	337.30	335.20	336.30
Water absorption at atmospheric pressure (%)	25.24	26.05	26.06	25.78

**Table 7:** Thermal conductivity of ecological mortar.

Standard	Test		*e,* Ws½/m²K	*λ,* W/mK
**EN 12664:2002**	Determination of Thermal Resistance	Sample 1	635.72	0.280
		Sample 2	627.74	0.270
		**Mean**	**631.73**	**0.275**

Thermal effusivity (***e***). Thermal conductivity (***λ***).

**Table 8:** Convective heat transfer coefficients of the building enclosures.

Position	Heat Flow	*R_so_,* m^2^ × K/W	*R_si_,* m^2^ × K/W
Vertical (façade)	Horizontal	0.040	0.130
Horizontal (ceiling)	Vertical and ascending	0.040	0.100
Horizontal (floor)	Vertical and descending	0.040	0.170

Outside convective heat transfer coefficient (***R_so_***). Inside convective heat transfer coefficient (***R_si_***).

**Table 9:** Solar absorptance values.

Building Enclosure	Surface	Color	Tone	*α*
Floor	Interior	Grey	Medium	0.65
Floor	External	Grey	Medium	0.65
Ceiling	Interior	White	Medium	0.30
Façade	External	Green	Dark	0.88
Façade	Interior	White	Medium	0.30
Interior partition	Interior	White	Medium	0.30

Solar absorptance (***α***).

**Table 10:** Linear thermal bridges.

Linear Thermal Bridges	*ψ,* W/m×K
Interior floor–façade	0.42
Exterior floor–façade	0.43
Projection corner	0.15
Entering corner	0.01
Window edge	0.24
Pillar–façade	0.84

Linear thermal transmittance. (***ψ***).

**Table 11:** Window features.

Material	*U,* W/m^2^ × K	*g*	*α*	*Afr/Aw,*%	*R_so_,* m^2^ × K/W	*R_si_,* m^2^ × K/W	*Q*_100_*,* m^3^/h × m^2^
Glazing	1.430	0.605	---	---	---	---	---
Frame	2.900	---	0.650	---	---	---	---
Glazing + frame	---	---	---	23.000	0.040	0.130	<3.000

Thermal transmittance (***U***). Solar factor (***g***). Solar absorptance (***α***). Area of a frame divided by area of a window (***Afr/Aw***). Outside convective heat transfer coefficient (***R_so_***). Inside convective heat transfer coefficient (***R_si_***). Permeability under a lab pressure of 100 Pa (***Q*_100_**).

**Table 12:** Set-point temperatures and mechanical ventilation in the user profile for the hospital inpatient ward floor.

**Days of the Year**	**Schedule—Set-Point Heating Temperature (low)**	***T,*** **°C**
Every day	0h00–23h00	20.00
**Days of the Week**	**Schedule—Set-Point Cooling Temperature (high)**	***T,*** **°C**
Every day	0h00–23h00	25.00
**Days of the Week**	**Schedule—Mechanical Ventilation**	**ren/h**
Every day	0h00–23h00	0.80

Temperature (***T***).

**Table 13:** Internal heat gains in the user profile for the hospital inpatient ward floor.

Owing to	Days of the Week	Schedule	*IHG,* W/m^2^
Sensible occupation	Every day	0h00–23h00	2.00
Latent occupation	Every day	0h00–23h00	1.26
Lighting	Every day	0h00–23h00	6.25
Equipment	Every day	0h00–23h00	1.50

Internal heat gains (***IHG***).

**Table 14:** Total internal heat gains in the user profile for the hospital inpatient ward floor.

Hours	0h00–23h00
***IHG,*** **W/m^2^**	11.01

Internal heat gains (***IHG***).

**Table 15:** Set-point temperatures and mechanical ventilation schedules of the user profile for the hospital outpatient consultation floor.

**Days of the Year**	**Schedule–Set-Point Heating Temperature (low)**	***T,*** **°C**
Working days	0h00–6h00 and 15h00–23h00	---
	7h00–14h00	20.00
Saturdays, Sundays and Holidays	0h00–23h00	---
**Days of the Week**	**Schedule—Set-Point Cooling Temperature (high)**	***T,*** **°C**
Working days	0h00–6h00 and 15h00–23h00	---
	7h00–14h00	25.00
Saturdays, Sundays and Holidays	0h00–23h00	---
**Days of the Week**	**Schedule—Mechanical Ventilation**	**ren/h**
Working days	0h00–6h00 and 15h00–23h00	---
	7h00–14h00	0.80
Saturdays, Sundays and Holidays	0h00–23h00	---

Temperature (***T***).

**Table 16:** Internal heat gains in the user profile for the hospital outpatient consultation floor.

Owing to	Days of the week	Schedule	*IHG,* W/m^2^
Sensible occupation	Working days	0h00–6h00 and 15h00–23h00	---
		7h00–14h00	6.00
	Saturdays, Sundays and Holidays	0h00–23h00	---
Latent occupation	Working days	0h00–6h00 and 15h00–24h00	---
		7h00–14h00	3.79
	Saturdays, Sundays and Holidays	0h00–23h00	---
Lighting	Working days	0h00–6h00 and 15h00–23h00	---
		7h00–14h00	6.25
	Saturdays, Sundays and Holidays	0h00–23h00	---
Equipment	Working days	0h00–6h00 and 15h00–23h00	---
		7h00–14h00	4.50
	Saturdays, Sundays and Holidays	0h00–23h00	---

Internal heat gains (***IHG***).

**Table 17:** Total internal heat gains in the user profile for hospital outpatient consultation floor on working days.

Hours	0h00–6h00	7h00–14h00	15h00–23h00
***IHG,* W/m^2^**	0.00	20.54	0.00

Internal heat gains (***IHG***).

**Table 18:** Monthly average air temperature in Burgos.

*T,* °C											
January	February	March	April	May	June	July	August	September	October	November	December
3.1	4.1	7.0	8.6	12.2	16.5	19.5	19.5	16.1	11.5	6.6	3.9

Temperature (***T***).

**Table 19:** Geometrical and thermophysical properties of the existing façade.

Material	*t,* m	*λ,* W/(m K)	*C_p_,* J/(kg K)	*δ,* kg/m^3^	*R_n_,* (m^2^ K)/W
Plasterboard	0.013	0.250	1000.000	825.000	---
Non-woven geotextile	0.010	0.060	1300.000	200.000	---
MW insulation	0.060	0.031	1000.000	40.000	---
Steel profiles	0.008	50.000	450.000	7800.000	---
MW insulation	0.020	0.041	1000.000	40.000	---
Ventilated air chamber	0.100	---	---	---	0.095
Slate cladding	0.040	2.200	1000.000	2400.000	---

Thickness (***t***). Thermal conductivity (***λ***). Specific heat (***C_p_***). Density (***δ***). Thermal resistance of a layer (***R_n_***).

**Table 20:** Geometrical and thermophysical properties of the façade with the ecological mortar block outward.

Material	*t,* m	*λ,* W/(m K)	*C_p_,* J/(kg K)	*δ,* kg/m^3^	*R_n_,* (m^2^ K)/W
Plasterboard	0.013	0.250	1000.000	825.000	---
Non-woven geotextile	0.010	0.060	1300.000	200.000	---
MW insulation	0.060	0.031	1000.000	40.000	---
Steel profiles	0.008	50.000	450.000	7800.000	---
MW insulation	0.020	0.041	1000.000	40.000	---
Ventilated air chamber	0.100	---	---	---	0.095
Ecological mortar block	0.100	0.275	1291.760	1058.000	---

Thickness (***t***). Thermal conductivity (***λ***). Specific heat (***C_p_***). Density (***δ***). Thermal resistance of a layer (***R_n_***).

**Table 21:** Geometrical and thermophysical properties of the façade with the ecological mortar block inward.

Material	*t,* m	*λ,* W/(m K)	*C_p_,* J/(kg K)	*δ,* kg/m^3^	*R_n_,* (m^2^ K)/W
Ecological mortar block	0.100	0.275	1291.760	1058.000	---
MW insulation	0.060	0.031	1000.000	40.000	---
Steel profiles	0.008	50.000	450.000	7800.000	---
MW insulation	0.020	0.041	1000.000	40.000	---
Ventilated air chamber	0.100	---	---	---	0.095
Slate cladding	0.040	2.200	1000.000	2400.000	---

Thickness (***t***). Thermal conductivity (***λ***). Specific heat (***C_p_***). Density (***δ***). Thermal resistance of a layer (***R_n_***).

**Table 22:** Features of the three types of façades.

Façade	*t,* m	*U,* W/(m^2^ K)	*w,* kg/m^2^
Existing	0.251	0.342	174.320
Exterior ecological mortar block layer	0.311	0.306	184.125
Interior ecological mortar block layer	0.328	0.326	267.400

Thickness (***t***). Thermal transmittance (***U***). Weight (***w***).

**Table 23:** Condensation on the current façade.

**Surface**		**Interstitial**							
***f_Rsi_ ≥ f_Rsi,min_***		***P_n_ ≤ P_sat,n_***	**Layer 1**	**Layer 2**	**Layer 3**	**Layer 4**	**Layer 5**	**Layer 6**	**Layer 7**
***f_Rsi_***	0.915	***P_sat,n_,*** **Pa**	754.453	785.198	963.519	963.583	2053.814	2185.170	2227.629
***f_Rsi,min_***	0.640	***P_n_,*** **Pa**	633.091	633.091	633.091	963.583	1088.379	1187.826	1285.323
**Material**		***t,*** **m**	***λ,*** **W/(m K)**	***µ***	***R_n_,*** **(m^2^ K)/W**	***U,*** **W/(m^2^ K)**	***P_vap_,*** **Pa**	***P_sat_,*** **Pa**	**Accumulated Condensation, ** **kg**
Slate cladding		4.0	2.2000	800	0.0182	55.0000	633.091	754.453	0.0000
Ventilated air chamber		10.0	1.0526	1	0.0950	10.5263	633.091	785.198	0.0000
MW insulation		2.0	0.0405	1	0.4938	2.0250	633.091	963.519	0.0000
Steel profiles		0.8	50.0000	1 × 10^15^	0.0002	6250.0000	963.583	963.583	2.4942
MW insulation		6.0	0.0310	1	1.9355	0.5167	1088.379	2053.814	0.0000
Non-woven geotextile		1.0	0.0600	5	0.1667	6.0000	1187.826	2185.170	0.0000
Plasterboard		1.3	0.2500	4	0.0520	19.2308	1285.323	2227.629	0.0000
**Totals**		25.1	---	---	2.9310	0.3420	---	---	---

Interior surface temperature factor (***f_Rsi_***). Minimum interior surface temperature factor (***f_Rsi,min_***). Vapor pressure of a layer (***P_n_***). Saturation pressure of a layer (***P_n,sat_***). Thickness (***t***). Thermal conductivity (***λ***). Factor of resistance to water vapor diffusion (***µ***). Thermal resistance of a layer (***R_n_***). Thermal transmittance (***U***). Vapor pressure (***P_vap_***). Saturation pressure (***P_sat_***).

**Table 24:** Condensations in the façade with the ecological mortar block outward.

**Surface**		**Interstitial**							
***f_Rsi_ ≥ f_Rsi,min_***		***P_n_ ≤ P_sat,n_***	**Layer 1**	**Layer 2**	**Layer 3**	**Layer 4**	**Layer 5**	**Layer 6**	**Layer 7**
***f_Rsi_***	0.924	***P_sat,n_,*** **Pa**	856.368	887.061	1062.757	1062.819	2082.175	2200.754	2238.940
***f_Rsi,min_***	0.640	***P_n_,*** **Pa**	633.091	633.091	633.091	1062.819	1149.123	1217.898	1285.323
**Material**		***t,*** **m**	***λ,*** **W/(m K)**	***µ***	***R_n_,*** **(m^2^ K)/W**	***U,*** **W/(m^2^ K)**	***P_vap_,*** **Pa**	***P_sat_,*** **Pa**	**Accumulated condensation, ** **kg**
Ecological mortar block		10.0	0.2750	5	0.3636	2.7500	633.091	856.368	0.0000
Ventilated air chamber		10.0	1.0526	1	0.0950	10.5263	633.091	887.061	0.0000
MW insulation		2.0	0.0405	1	0.4938	2.0250	633.091	1062.757	0.0000
Steel profiles		0.8	50.0000	1 × 10^15^	0.0002	6250.0000	1062.819	1062,819	1.5371
MW insulation		6.0	0.0310	1	1.9355	0.5167	1149.123	2082.175	0.0000
Non-woven geotextile		1.0	0.0600	5	0.1667	6.0000	1217.898	2200.754	0.0000
Plasterboard		1.3	0.2500	4	0.0520	19.2308	1285.323	2238.940	0.0000
**Totals**		31.1	---	---	3.2770	0.306	---	---	---

Interior surface temperature factor (***f_Rsi_***). Minimum interior surface temperature factor (***f_Rsi,min_***). Vapor pressure of a layer (***P_n_***). Saturation pressure of a layer (***P_n,sat_***). Thickness (***t***). Thermal conductivity (***λ***). Factor of resistance to water vapor diffusion (***µ***). Thermal resistance of a layer (***R_n_***). Thermal transmittance (***U***). Vapor pressure (***P_vap_***). Saturation pressure (***P_sat_***).

**Table 25:** Condensations in the façade with the ecological mortar block inward.

**Surface**		**Interstitial**							
***f_Rsi_ ≥ f_Rsi,min_***		***P_n_ ≤ P_sat,n_***	**Layer 1**		**Layer 2**	**Layer 3**	**Layer 4**	**Layer 5**	**Layer 6**
***f_Rsi_***	0.919	***P_sat,n_,*** **Pa**	753.582		782.824	951.557	951.616	1962.311	2232.679
***f_Rsi,min_***	0.640	***P_n_,*** **Pa**	633.091		633.091	633.091	951.616	988.889	1285.323
**Material**		***t,*** **m**	***λ,*** **W/(m K)**	***µ***	***R_n_,*** **(m^2^ K)/W**	***U,*** **W/(m^2^ K)**	***P_vap_,*** **Pa**	***P_sat_,*** **Pa**	**Accumulated Condensation, ** **kg**
Slate cladding		4.0	2.2000	800	0.0182	55.0000	633.091	753.582	0.0000
Ventilated air chamber		10.0	1.0526	1	0.0950	10.5263	633.091	782.824	0.0000
MW insulation		2.0	0.0405	1	0.4938	2.0250	633.091	951.557	0.0000
Steel profiles		0.8	50.0000	1 × 10^15^	0.0002	6,250.0000	951.616	951.616	0.7516
MW insulation		6.0	0.0310	1	1.9355	0.5167	988.889	1962.311	0.0000
Ecological mortar block		10.0	0.2750	5	0.3636	2.7500	1285.323	2232.679	0.0000
**Totals**		32.8	---	---	3.0760	0.3260	---	---	---

Interior surface temperature factor (***f_Rsi_***). Minimum interior surface temperature factor (***f_Rsi,min_***). Vapor pressure of a layer (***P_n_***). Saturation pressure of a layer (***P_n,sat_***). Thickness (***t***). Thermal conductivity (***λ***). Factor of resistance to water vapor diffusion (***µ***). Thermal resistance of a layer (***R_n_***). Thermal transmittance (***U***). Vapor pressure (***P_vap_***). Saturation pressure (***P_sat_***).

**Table 26:** Thermal inertia of the façades.

Façade	*I,* J/(m^2^ × K × s^1/2^)	*m_t_,* J/(m^2^ × K)	*CTT,* *s*	*m_tu_,* J/(m^2^ × K)	*% m_tu_,* %
Current	16,199.977	140,605.000	63,775.714	21,756.66	15.47
With the ecological mortar block outward	14,515.208	181,273.208	104,787.480	31,978.85	17.64
With the ecological mortar block inward	16,233.985	263,948.208	404,894.150	131,617.71	49.86

Thermal inertia (***I***). Thermal mass (***m_t_***). Thermal constant (***CTT***). Useful thermal mass (***m_tu_***). Useful thermal mass percentage (***%m_tu_***).

**Table 27:** Yearly heating and cooling energy demands.

Hospital Floor	Façade	Heating Energy Demands		Cooling Energy Demands	
		kWh/Year	kWh/(m^2^ × Year)	kWh/Year	kWh/(m^2^ × Year)
Hospital inpatient ward floor	Existing	161,986.27	94.71	15,053.79	8.80
	Exterior ecological mortar block	161,217.55	94.26	14,969.17	8.75
	Interior ecological mortar block	160,674.54	93.94	14,091.91	8.24
Hospital outpatient consultations floor	Current	54,462.48	62.32	8171.31	9.35
	Exterior ecological mortar block	54,198.18	62.02	8242.97	9.43
	Interior ecological mortar block	54,878.39	62.80	8209.38	9.39
